# NETome: A model to Decode the Human Genome and Proteome of Neutrophil Extracellular Traps

**DOI:** 10.1038/s41597-022-01798-1

**Published:** 2022-11-16

**Authors:** David Scieszka, Yi-Han Lin, Weizhong Li, Saibyasachi Choudhury, Yanbao Yu, Marcelo Freire

**Affiliations:** 1 Department of Genomic Medicine and Infectious Diseases, J. Craig Venter Institute, La Jolla, CA 92037 USA; 2 Department of Infectious Diseases, J. Craig Venter Institute, Rockville, MD 20850 USA; 3 Division of Infectious Diseases and Global Public Health, University California San Diego, La Jolla, USA; 4 Present Address: National Center for Advancing Translational Sciences, Rockville, 20850, Maryland USA; 5 Present Address: Department of Chemistry and Biochemistry, University of Delaware, Newark, 19716 Delaware USA

**Keywords:** Immunology, Biomarkers

## Abstract

Neutrophils are the most abundant type of white blood cells in humans with biological roles relevant to inflammation, and fighting off infections. Neutrophil Extracellular Traps (NETs) act as enxogenous agents controlling invasion by bacteria, viruses, fungi, metabolic, and traumatic agents. Traditionally, studies have focused on elucidating molecular and cellular pathways preceding NET formation. Here, we developed a model to decode the human genome and proteome of developted NETs. Via *in vitro* system to differentiate HL-60 human myeloid cell line into neutrophil extracellular trap (ecTrap) producing cells, we isolated and captured ectrap derived DNA and proteins for shotgun sequencing. The genomic sequences revealed accurate delineation of gene composition including immune response genes and mitochondrial enrichment, while providing a reference database for future interrogation. Shotgun proteomics showed global proteins in differentiated cells with specific immune pathways when compared to undifferentiated counterparts. Coupled with omics’ approaches, we validated our system by functional assays and began to dissect host-microbial interactions. Our work provides a new understanding of the genomic and proteomic sequences, establishing the first human database deposition of neutrophil extracellular traps.

## Abstract (summary)

**Table:** 

Measurement(s)	Genome Sequence Reads • Protein Abundance
Technology Type(s)	Illuminia • liquid chromatography-tandem mass spectrometry
Factor Type(s)	Neutrophil extracellular trap genome sequence • Neutrophil extracellular trap protein abundance
Sample Characteristic - Organism	Homo sapiens
Sample Characteristic - Environment	cell culture

## Background & Summary

As polymorphonuclear leukocytes of the phagocytic system, neutrophils are essential for early immune defense in patrolling the human body in health and in sites of infection to fight infectious agents^1^. Neutrophils generated from myeloid precursors in the bone marrow prior to differentiating into several stages of maturation, including myeloblast, promyelocyte, myelocyte, metamyelocyte, bands, and polymorphonuclear cells. Neutrophils are the most abundant types of white blood cells in mammals - approximately 100 billion are produced in the human body every day. From the blood circulation, neutrophils are recruited by chemical and physical cues to the periphery by infiltration of tissues and interstitial areas to detect and eliminate threats. Upon localization, neutrophils present key functions in the clearance of chemical and pathogens such as bacteria^2^, fungi^3^, viruses^4^, and parasites^5^. In addition to the early recruitment to infection sites and clearance mechanisms by phagocytosis, oxidative burst, and degranulation, another much less recognized means of extracellular attack can develop.

Web-like chromatin structures known as neutrophil extracellular traps (NETs) are produced and ejected into the extracellular space for host protection and infection control^6,7^. To accomplish microbial clearance, the DNA backbone of NETs is attached to molecules, such as histones, calprotectin, and cathepsin G protease, which provide antimicrobial properties to eliminate invaders^8^. Although NETs are intended for host protection, neutrophil response and NET production require a fine balance^9^,^10^. Namely, underactivity leads to increased invasion from pathogens, whereas over-activity is highly damaging to tissues. This secondary damage to tissue from sustained formation can lead to a cascade of inflammatory reactions, resulting in organ damage, cancer, tissue loss and thrombosis. When dysregulated, excessive NET release has been implicated in severe disease states, including lupus^11^, COPD^12^, type 2 diabetes^13^, chronic inflammation^14^, cystic fibrosis^15^, autoimmunity^16^, and cancers^17^, among others^18,19^.

Originally, it was thought that the release of DNA into the extracellular environment was not regulated, and that molecules involved were randomly adhered. However, it is now accepted that this release is a fine-tuned and well-controlled intracellular process. Many factors are now known to guide the generation of NETs, including neutrophil elastase (NE), peptidyl arginine deiminase type 4 (PADI4), and gasdermin D. Furthermore, extensive research has shown that NET release can differ based on the type, concentration, and duration of stimulus presented^3,7,17,19–21^. More broadly, stimuli from microbes and chemicals act differently in activation, cell membrane rupture, and NET expulsion. In response to a more replicable substance, phorbol 12-myristate 13-acetate (PMA) activates common intracellular pathways in neutrophils, including protein kinase C (PKC)-mediated pathways, and MAPK/ERK signalling which generates downstream reactive oxygen species (ROS) via myeloperoxidase (MPO). MPO utilizes ROS to mediate the activation of NE which translocate into the nucleus for initial histone degradation and chromatin unpacking. MPO further promotes chromatin unpacking by activating PADI4, which is responsible for the citrullination of histones. Despite the current understanding, there are multiple molecules released from neutrophils that are yet unknown, highlighting the need to decode the exact sequences of the human NETs.

With emerging interest in neutrophil biology and phenotypic characterization, the lack of models limits the use of NETs in experimental research. Mainly, these limitations are based on inherent neutrophil properties, such as a brief lifespan, high sensitivity to handling and temperature. Collectively, these inherent properties make it challenging to achieve replicability. To revisit these issues and determine NET composition, we devised an *in vitro* model, developing a protocol that reliably produced facsimile NETs in a sterile inflammation setting (see table Table 1 for complete list of materails). We leveraged a myeloid undifferentiated cell line (HL-60 cell line) into cell differentiation (dHL60) to produce NET for interrogations. Through concentration-dependent and time point PMA perturbation studies, we successfully transformed dHL60 cells into ecTrap producing cells. Here, we aimed to identify the genomic sequence of NET and to characterize their molecular contents (specific proteins, and metabolic markers for NET scaffold-ecTraps). Although these are not bona fide NETS, we demonstrated through imaging, staining, sequencing, and bacterial clearance that the sequences were comparable to viable NET materials. After validation, whole-genome sequencing allowed for the comparison of sequences to undifferentiated myeloid HL-60 and dHL60. Finally, we compared our data with published datasets to understand the replicability of our model to other neutrophil models. Our study showed a replicable model system to produce ecTraps and identified the genomic and proteomic contents of the differentiated HL-60 cells. This first human sequence study deposits the exact content material into freely accessible databases and opens new avenues for elucidating NETomic structures.

**Table 1:** List of Reagents and Platforms.

Reagent/Item Description	Reference number
HL-60 cell line	ATCC, cat. no. CCL-240
IMDM	Gibco, cat. no. 12440061
FBS	ATCC, cat. no. 30–2020
Penicillin + Streptomycin	Gibco, cat. no. 15140122
Cell Imaging Cytometer	Nexcelom, CeligoS, cat. no. 200-BFFL-S
Agilent Bioanalyzer 2100	Agilent, model G2939A
QuBit 2.0	Invitrogen, cat. no. Q32866
Qiagen AllPrep DNA/RNA/Protein Minikit	Qiagen, cat. no. 80004
Ultracentrifuge tube	cat. no. 149569 C
On-Chip Sort Flow Cytometer-2D Chip-Z1001	cat. no. 1002004
MitoSOX™ Red Mitochondrial Superoxide Indicator	Invitrogen, cat. no. M36008, lot 2015529
Eosin Y, 0.25% (w/v) in 57% Alcohol	Ricca Chemical, cat. no. 284516
Hematoxylin Stain Solution, Gill 1 formulation, regular strength	Ricca Chemical, cat. no. 353516
Giemsa Stain Solution	LabChem, Inc., cat. no. LC148407
Confocal Microscope	Leica TCS SP5 II
Cytofix/Cytoperm Fixation and Permeabilization solution	BD Biosciences, cat. no. 55472251-2090KZ
DAPI	Invitrogen, cat. no. D1306
Cytoseal-60 mounting media	Thermo Scientific, cat. no. 83104
Tomographic Microscope	Nanolive 3D Cell Explorer
Scanning Electron Microscope	Leica UCT Ultramicrotome
Grid Viewing Microscope	Tecnai G2 Spirit BioTWIN transmission electron microscope equipped with an Eagle 4k HS digital camera (FEI)
Formvar-carbon-coated copper grids	100 mesh, Electron Microscopy Sciences, Hatfield, PA) uranyl acetate (Ladd Research Industries, Williston, VT
Grid Examination	EOL JEM-1400Plus transmission electron microscope operating at 80 kV
Grid Recording Camera	Gatan OneView 4 K
NexteraXT library prep kit	Illumina, cat. no. FC-131-1096
Sequencing Platform	Illumina, NovaSeq 6000
Flow Cell	S2 2X150bp
DNA Mapping Software	CLC Workbench v11, v12
Neutrophil Elastase ELISA	Abcam, cat. no. ab204730

## Methods

### Experimental design

To achieve our goal of creating an *in vitro*, facsimile-NET pipeline, promyeloblast human cell line HL-60 cells were selected due to their ability to differentiate into cells with neutrophil characteristics (morphology, phagocytosis, chemotaxis, etc.). Kinetic studies were performed to validate our model against the previously published timeline of differentiation, and different concentrations of PMA that were required to induce optimal ecTrap release. These were validated through visual inspection, immunofluorescence, MitoSOX-red assays, NanoDrop, flow cytometry, and Qubit.

### HL-60 cell culture

Promyeloblast human cell line, HL-60, was acquired from ATCC. HL-60 cell lines were maintained in culture media prepared according to manufacturer’s guidelines. Briefly, the HL-60 culture media consisted of Iscove’s modified Dulbecco’s medium (IMDM, Gibco, cat. no. 12440-061) with 5% fetal bovine serum (FBS, heat inactivated, Gibco, cat. no. 26140-095) and 1X antibiotic (Penicillin + Streptomycin; Gibco, cat. no. 15-140-122). Note: Avoid using antimycotic in the recovery media as it affects the recovery and growth of HL-60 cell lines. Cell culture medium, FBS and cell lines were tested for mycoplasma contamination prior and after arrival. Our facilities (incubator, sterile hoods, etc.) are often checked for mycoplasma regularly.

### Differentiation of HL-60 to neutrophils (dHL60)

HL-60 cells in culture media were centrifuged at 275 × g for 10 minutes at room temperature (RT) and the culture media was aspirated. Cell pellets were resuspended in differentiation media (i.e., culture media containing 1.5% dimethyl sulfoxide) to an initial cell seed count of 10E5 cells. Verification of a previous neutrophil differentiation timeline was performed^22^. To quantitatively determine the maximum number of differentiated cells before apoptosis, a growth curve was created using a 96-well plate measured daily for 5 days (Fig. S7). Morphological changes were monitored by Giemsa staining through the comparison of our *in vitro* model HL-60 cells to differentiated HL-60 cells (dHL60) i.e., neutrophils.

### Neutrophil extracellular Trap model production with dHL60 Cell line

**Fig. 1:**
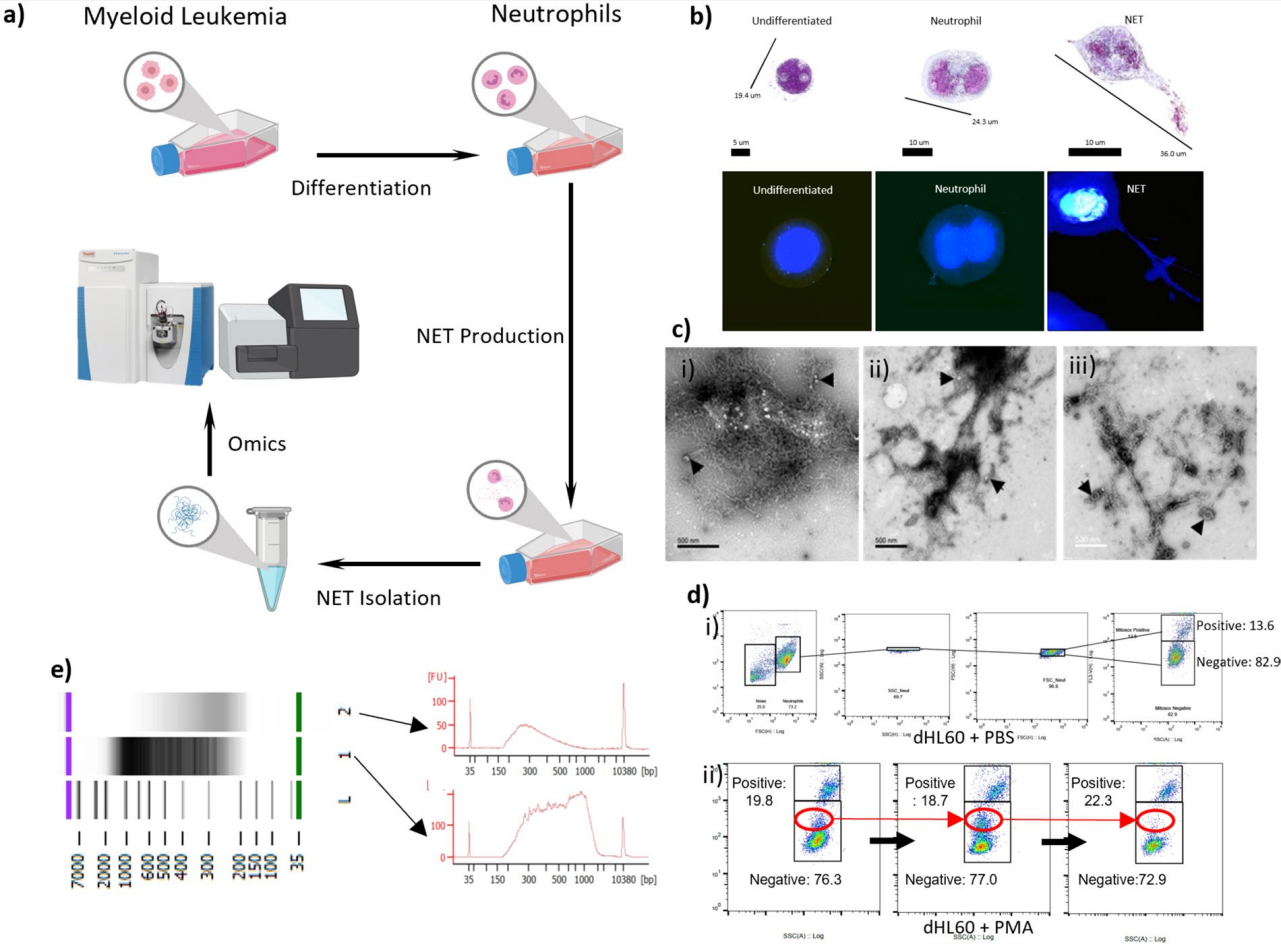
Neutrophil Extracellular Trap Production and Isolation. (**a**) Schematic of *in vitro* ecTrap production. Cultured HL-60 cell lines are incubated with DMSO to differentiate into neutrophils (dHL60 cells). After which, PMA stimulation leads to ecTrap production, isolation, and “omics” analysis. (**b**) Representative 3D holotomographic microscopy images digitally stained based on RI (refractive index) confirm the differentiation of HL-60 cells to neutrophils (dHL60) after 4 days in differentiation media, and successful release of ecTraps after 4-hour incubation in 1,000 nM PMA. (**c**. i-iii) Scanning electron microscope (SEM) images of isolated DNA samples from (i) HL-60 cells, (ii) dHL60 cells and (iii) released ecTrap of static cells. All samples show the presence of lipid bilayers (indicated by arrowheads). (**d**) Mitochondrial superoxide generation before - and during - ecTrap release process was measured by MitoSOX assay over 5 hours of incubation with PMA. (i) Doublet discrimination gating strategy was used to ensure accurate MitoSOX-red quantification. Panels shown are negative control (dHL60 in PBS) (ii) Representative panel of flow cytometry analysis shows the generation of superoxide in dHL60 on incubation with PMA over 0.5 hours. Red circle highlights a population shift from MitoSOX negative to MitoSOX positive. (**e**) DNA quantification by Agilent High-sensitive DNA chip verifies the composition of extracted ecTrap samples. Lane 1 shows an isolated ecTrap DNA sample. Lane 2 shows an isolated ecTrap sample after incubation with DNase to digest all DNA contents. Arrows indicate the electropherogram of each sample in the gel image above. Lane L shows the DNA ladder marker.

Multiwell plates were used in kinetic studies to determine effective ecTrap release from dHL60 cells. PMA was used in this *in vitro* model to induce ecTrap release at concentrations ranging from 0.1 nM - 10,000 nM and at time points ranging from 10 minutes to 6 hours. Morphological changes were monitored through different microscopy techniques (Figs. 1b,c, S1A) and DNA was quantified by NanoDrop spectrophotometer, Agilent High-sensitivity DNA chip using Bioanalyzer 2100, and QuBit 2.0.

### DNA Isolation from HL-60, dHL60, and neutrophil extracellular traps

HL-60 and dHL60 DNA was isolated via the Qiagen AllPrep DNA/RNA/Protein Mini Kit according to the manufacturer’s protocol^23^ and suspended in an elution buffer (EB) for storage at −20 °C. DNA purity was quantified with NanoDrop spectrophotometer, QuBit 2.0, and Agilent High-sensitivity DNA chip (Fig. 1e).

We adopted a previous protocol for NET production from primary blood cells^24^. After 4 hours of incubation in PMA, culture media was gently aspirated to remove non-ecTrap forming cells. Adherent, ecTrap-releasing cells were resuspended in ice-cold PBS (-calcium, -magnesium) to a final volume of 12 mL. The solution was centrifuged at 275 × g for 10 minutes to pellet cells. The DNA-rich supernatant was transferred to an ultracentrifuge tube and the cell pellet was discarded. Ultracentrifugation was performed at 18,000 × g for 10 minutes at RT to pellet DNA and the PBS (-) supernatant was aspirated. DNA pellets were resuspended in EB for storage at −20 °C.

### MitoSOX staining and flow cytometry

The On-Chip Sort flow cytometer was used to quantify dHL60 ROS generation by staining with MitoSOX. Multiwell plates were used to compare HL-60 and dHL60 cells in PBS (-) and in PMA over the course of 5 hours at 30-minute increments. In total, three experimental replicates were performed and analysed (Fig. S1B).

### Cell staining and microscopy

To monitor nuclear morphological changes, HL-60 and dHL60 cells were stained with Eosin Y, Haematoxylin and Giemsa according to conventional methods and viewed under a histological microscope. Fluorescent staining was visualized using a Leica TCS SP5 II confocal microscope to qualify ecTrap DNA release when compared to *E. coli* co-incubation. Both groups were permeabilized, stained with DAPI (1,000 nM), fixed with 4% PFA for 15 minutes, and checked for fluorescence^25^. Additionally, nuclear morphology changes and ecTrap release were visualized via 3D-Cell Explorer Nanolive microscope under fixed, and unfixed conditions (Figs. 1b, S1A). Comparative visualizations of isolated HL-60, dHL60, and ecTrap DNA was further performed using scanning electron microscopy (Fig. 1c). Neutrophil elastase (NE) fluorescence and growth curves were monitored using the Celigo S cell imaging cytometer. Briefly, coculture experiments with *Fusobacterium* were performed in 6-well plates at a 1:10 ratio of dHL60 to bacteria, respectively (Fig. S1C). Standard curves were generated according to manufacturer instructions. Celigo internal cell counting software was utilized to determine cell growth each day over 5 days to determine cell division and terminal differentiation in 6-well plates (Fig. S7).

### Scanning electron microscopy

HL-60, dHL60, and ecTrap DNA samples were retrieved from −20 °C and allowed to thaw on ice. Once thawed, samples were dehydrated in ethanol, embedded in epoxy resin, sectioned at 50–60 nm on a Leica UCT ultramicrotome, and picked up on Formvar and carbon-coated copper grids. Sections were stained with 2% uranyl acetate for 5 min and Sato’s lead stain for 1 min. Grids were viewed using a Tecnai G2 Spirit BioTWIN transmission electron microscope equipped with an Eagle 4k HS digital camera (FEI).

Formvar-carbon-coated copper grids (100 mesh, Electron Microscopy Sciences, Hatfield, PA) were placed on 20 μl drops of each sample solution displayed on a Parafilm sheet. After allowing material to adhere to the grids for 10 minutes, grids were washed 3 times by rinsing through 200 μl drops of milli-Q water before being left for 1 min on 2% (wt./vol.) uranyl acetate (Ladd Research Industries, Williston, VT). Excess solution was removed with Whatman 3 M blotting paper, and grids were left to dry for a few minutes before viewing. Grids were examined using a JEOL JEM-1400Plus transmission electron microscope operating at 80 kV. Images were recorded using a Gatan OneView 4 K digital camera (Fig. 1c).

### Protein isolation

**Fig. 2:**
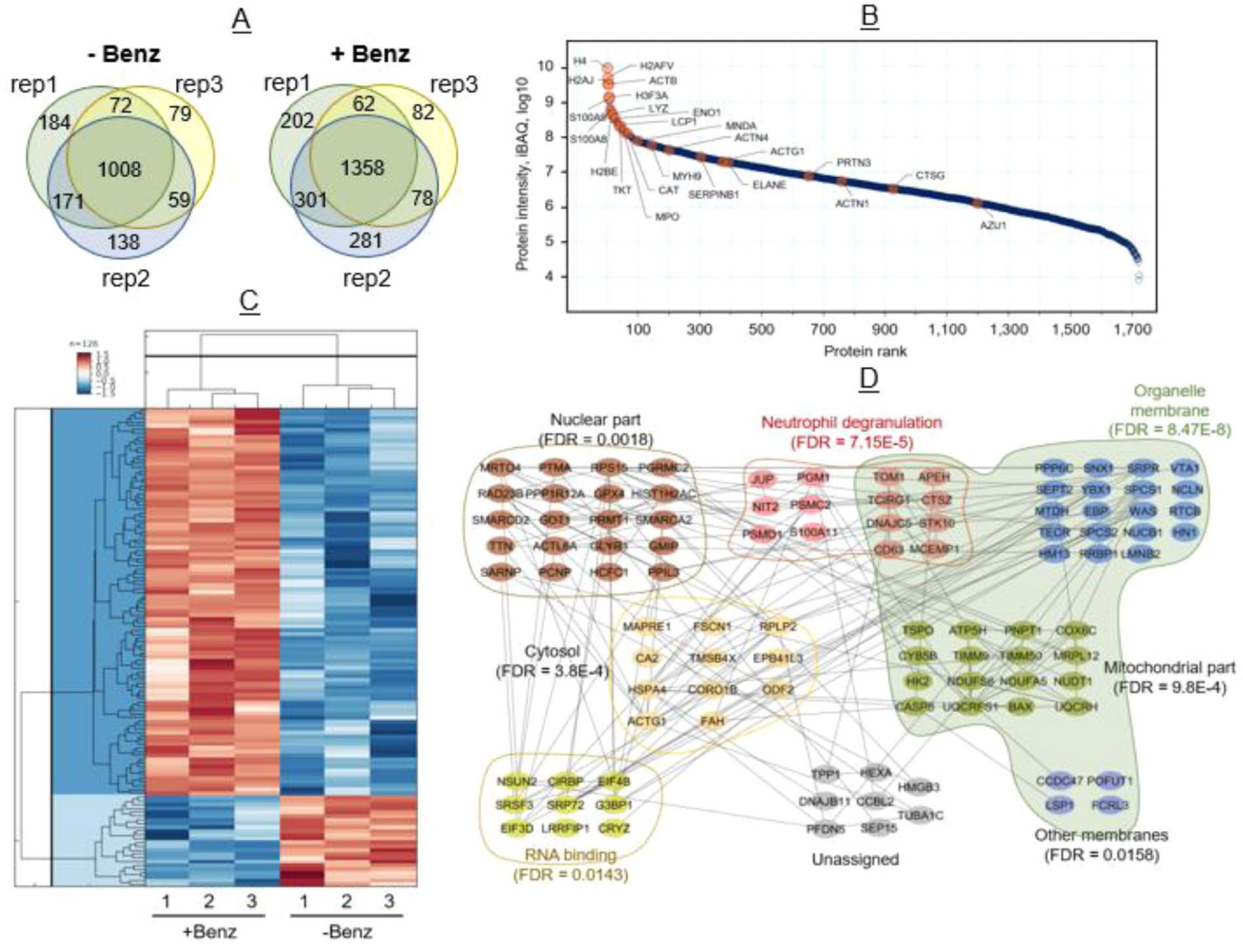
dHL60 induced ecTrap Proteome. (**a**) Proteome analysis of ecTrap from three representative samples (i.e., rep1, rep2, and rep3) identified a total of 2,364 proteins after Benzonase treatment and 1,711 proteins in untreated samples. Common proteins found among three representative samples in Benzonase treated and untreated ecTrap is 1,358 and 1,008, respectively. (**b**) Dynamic range of the ecTrap proteome. Data showing (1,722 proteins) here is from Benzonase-treated ecTrap. Median values of the three replicate experiments were used for the plot. Previously reported proteins associated with NET by Urban *et. al*. denoted by orange dots, most of which ranked among the 100 most abundant proteins found in our ecTrap samples. (Median value of three experimental replicates are plotted here). **(c)** Hierarchical clustering of the 126 significant proteins (fold change ≥2 or ≤−2; Permutation FDR 0.05) between the two groups. Z-scored LFQ intensities were color-coded as indicated in the scale bar. **(d)** STRING protein network and Gene Ontology analysis of 101(out of 126) significantly enriched proteins in ecTraps after Benzonase treatment were analysed using embedded STRING app in CytoScape software (version 3.7.2). The confidence score cut-off was set to 0.4. Representative enriched gene ontology (GO) terms (e.g., biological process, molecular function, and cellular compartment) and corresponding FDR values were depicted in the network.

Isolation of HL-60 and dHL60 protein was conducted via the Qiagen AllPrep DNA/RNA/Protein Mini Kit and performed according to the manufacturer’s instructions^23^. Attempts using the AllPrep to isolate protein from total ecTrap samples were unsuccessful and the total ecTrap isolation protocol was used for proteomics analysis (Fig. 2, S4, S5, S6).

### Neutrophil Extracelular Trap protein preparation for proteomics

After thaw from −80 °C, ecTrap solutions were added with protease inhibitors and 1% Benzonase Nuclease (Sigma) and incubated at 37 °C for 20 min to remove nucleic acids. Protein samples were then processed using the Suspension Trapping (STrap) approach as described previously^26^ to generate tryptic peptides for liquid chromatography-tandem mass spectrometry (LC-MS/MS) analysis. To specifically identify nucleic acid-bound proteins, one set of ecTrap samples were processed similarly but excluded the Benzonase treatment.

In total, LC-MS/MS was performed on 6 protein samples (three ecTrap with Benzonase treatment and three ecTrap without Benzonase treatment) following a protocol described previously^27^. In brief, the desalted samples were first resuspended into 20 μl 0.1% formic acid in water and then loaded onto a trap column (2 cm × 300μm, PepMap C18, Thermo Scientific) and an analytical column (19 cm × 75μm, 3.0 μm; ReproSil-Pur C18-AQ media) coupled to an Ultimate 3000 nano-LC and Q-Exactive mass spectrometer system (Thermo Scientific). Protein identification and quantitation were performed using Proteome Discoverer (version 2.2) and MaxQuant-Perseus software suite. The UniProt human database (20,413 sequences, reviewed only; version 2018_12) was used for protein search. Only the peptide and protein identifications with false discovery rate (FDR) of 1% or less were accepted in the final data set. More details of proteomic procedures can be found in the recent publication^27^. The mass spectrometry proteomics data have been deposited to the ProteomeXchange Consortium via the PRIDE partner repository with the dataset identifier PXD016143^28^.

### Neutrophil Extracelular Trap genomic sequencing

Illumina’s NexteraXT library prep kit was used for HL-60, dHL60, and ecTrap samples and sequenced on the NovaSeq 6000 platform using an S2 flow cell 2X150bp. 400pM of each sample pool was loaded and 1% PhiX was spiked in each lane^29^. Cluster density was 2961 K/mm2 with 80% PF. 1429.42 Gb and 4.5B PE Reads were generated. Coverage for the HL-60, dHL60, and ecTrap samples were 36X, 45X, and 47X respectively. The raw genomic sequences are available at NCBI Sequence Read Archive (SRA) under BioProject under accession PRJNA587717 at https://www.ncbi.nlm.nih.gov/bioproject/PRJNA587717.

The resultant fastq files were input into the CLC Workbench (v11), trimmed by 15 nt on the 3′ end to remove primers, trimmed for q-scores < 25, mapped to the human genome (hg38, CLC v12), and checked for normal human GC content. To determine whether the deposited sequence data was from ecTrap, a sliding window analysis was conducted for every 20, 100, 500, and 5,000 nt. Regions of interest (ROIs) were selected based on known protein association^8^, proteomics results, and nonselective general analysis. Sliding window files of aforementioned sizes are available through the corresponding author. Additionally, unmappable read files are also available and were not used in our analysis.

### Normalization steps for genomics sliding window

Normalization of sliding window coverage was performed by dividing coverage per sliding window by complete coverage per sample per chromosome.

$${\rm{E}}{\rm{.g}}.:\frac{5,000bp\;Window\;Coverage\;for\;NET\;on\;Chromosome\;1}{Total\;Sample\;Coverage\;for\;NET\;on\;Chromosome\;1}$$

Normalization of mitochondrial DNA was performed by dividing coverage per sliding window by complete coverage per sample for the entire genome.

$${\rm{E}}{\rm{.g}}.:\frac{20bp\;Window\;Coverage\;for\;NET\;on\;Chromosome\;MT}{Total\;Sample\;Coverage\;for\;NET\;Chromosomes}$$

### Known protein association

Proteins known to be localized within NETs were tested for genomic enrichments or depletions based on normalized 20nt sliding windows^8^. These proteins of interest (POIs) were input into Ensembl biomart (Release 96) and their genomic start/end locations were determined.

### Proteomic protein expression selection

Proteomics analysis was used to retroactively determine ROIs. The genomic regions associated with the protein results were input into *Ensembl* biomart and their genomic start/end locations were determined. Enrichment/depletion analysis was performed with 20 nt normalized sliding windows.

### Nonselective general analysis used for comparison

Using the 5,000 nt normalized window analysis, regions were selected based on 1.5-fold-change for each sample compared to the others simultaneously or compared to the average of the other two samples. The annotated list used for exonic analysis was acquired from *Ensembl* biomart and used in conjunction with the normalized 5,000nt windows. Overlapping expression analysis was performed by downloading the neutrophil gene expression data table (http://collinslab.ucdavis.edu/neutrophilgeneexpression/)^30^ and comparing the gene list generated from our nonselective general analysis. The initial range of genomic coverage spanned several orders of magnitude, and a percentage of the total became more representative for visualization. Namely, each sample was divided by the average of all three samples.

$${\rm{E}}{\rm{.g}}{\rm{.:}}\,\frac{NET\;GeneX\;Coverage}{\left(\frac{NET\;GeneX+HL60\;GeneX+dHL60\;GeneX}{3}\right)}$$

**Fig. 3:**
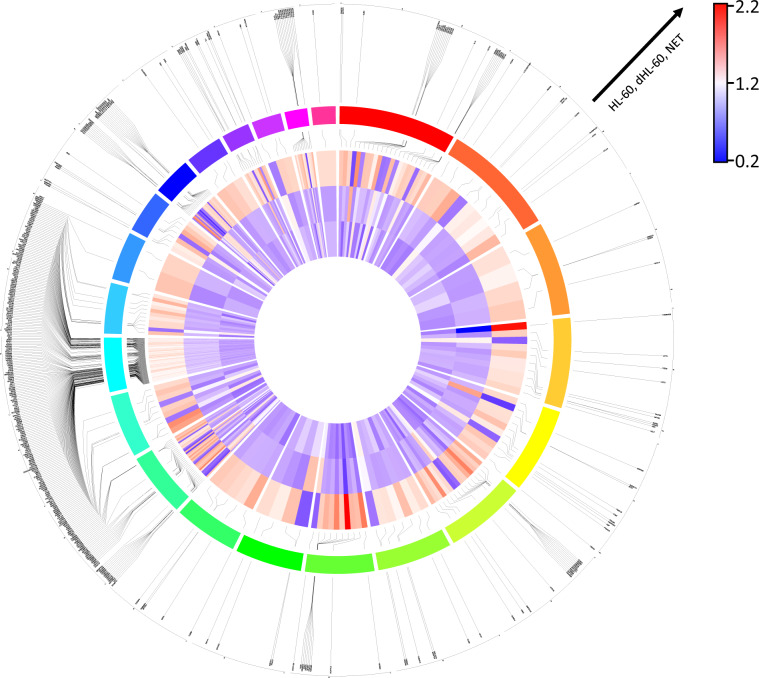
Gene Enrichment/Depletion Comparison Published Database. A 1.5-fold cut-off enrichment screen was used to determine regions of enrichment/depletion. Using annotated gene coding regions, a comparison to published expression data was used to determine overlap. The resultant circos plot for chromosomes 1–22 is shown. From innermost to outermost tracks: heatmap in order of HL-60, dHL60, and ecTrap enrichments; linkers from heatmap to color coded chromosomes in order from red (chromosome 1) clockwise to pink (chromosome 22); and linkers to gene names. Table of gene names used is available upon request.

It should be noted that this normalization was simply for aesthetic reasons in order to visualize that our ecTrap sequence was different from the HL-60 and dHL60 sequences (Fig. 3) and was not performed during our initial genome-wide scan. All circos plots were generated using the ShinyCircos software (http://shinycircos.ncpgr.cn/).

Statistical significance was calculated with a nonparametric Mann-Whitney test in the Prism software (Graphpad Software Inc.). Statistical significance for proteomic dataset was calculated with ANOVA. Genomic statistical significance for ecTrap, telomeric frequency, and mitochondrial enrichments were calculated using ANOVA. Statistical significance for correlations was calculated by Spearman rank test with P values and r values noted on the respective graphs. *p < 0.05, **p < 0.01, ***p < 0.001, and ****p < 0.0001 were considered significant and are referred to as such in the text.

## Data Records

The mass spectrometry proteomics data have been deposited to the ProteomeXchange Consortium via the PRIDE partner repository with the dataset identifier PXD016143^28^. The raw whole genomic sequence reads are available from NCBI Sequence Reads Achieve (SRA) under BioProject id: PRJNA587717. Sliding window files can be referenced as follows.

### ecTraps

NCBI^31^ https://sra-downloadb.be-md.ncbi.nlm.nih.gov/sos2/sra-pub-run-17/SRR1039804/SRR10398045.1

AWS https://sra-pub-run-odp.s3.amazonaws.com/sra/SRR10398045/SRR10398045

GCP gs://sra-pub-run-8/SRR10398045/SRR10398045.1

### dHL-60

NCBI^32^ https://sra-downloadb.be-md.ncbi.nlm.nih.gov/sos2/sra-pub-run-17/SRR1039804/SRR10398046.1

AWS https://sra-pub-run-odp.s3.amazonaws.com/sra/SRR10398046/SRR10398046

GCP gs://sra-pub-run-8/SRR10398046/SRR10398046.1

### HL-60

NCBI^33^ https://sra-downloadb.be-md.ncbi.nlm.nih.gov/sos2/sra-pub-run-17/SRR1039804/SRR10398047.1

AWS https://sra-pub-run-odp.s3.amazonaws.com/sra/SRR10398047/SRR10398047

GCP gs://sra-pub-run-8/SRR10398047/SRR10398047.1

### Morphological and growth data

Data are hosted on figshare and accessed using the following links:

Main File: https://figshare.com/projects/NETome/128357

DAPI Staining^34^: 10.6084/m9.figshare.17161160.v2

MitoSox Dataset^35^: 10.6084/m9.figshare.17156261.v1

Giemsa Staining^36^: 10.6084/m9.figshare.17161142.v1

Growth Curve Dataset^37^: 10.6084/m9.figshare.17156195.v1

### Proteomic data

Proteomic data has been submitted to the PRIDE database under the accession PXD016143^28^.

## Technical Validation

### dHL60 cell differentiation

To develop a dHL60 differentiation protocol and produce NET-like ecTraps *in vitro*, we first established a differentiation system that allowed cells to be viable with synchronized ecTrap production. Extensive literature related to HL-60 cells have made this an attractive model for studies of differentiation. However, neutrophil differentiation protocols lead to increased cell death. Thus, we established a threshold of differentiation that would keep viability at 80% (Fig. S7A). HL-60 cells grow in suspension culture with a doubling time that can vary from 20–45 hrs. Morphologically, the cells present with rounded nuclei and basophilic cytoplasm with azurophilic granules (Figs. 1b, S1A). We initiated testing cell differentiation by culturing undifferentiated HL-60 cells with culture medium enriched for DMSO (0.1–10%). As compared to steady state, HL-60 differentiation led to cytoplasm enlargement, nuclear condensation, and segmentation. We evaluated to confirm cell morphology histochemically through trypan blue, Giemsa, and by flow cytometry analysis of CD11b, as it represents a marker for early differentiation. We then established dHL60 differentiation at the viability threshold, with morphological characteristics and surface marker validation at 4 days under 1.5% DMSO (Figs. 1b, S1A, S7B). As expected, cells were terminally differentiated and presented phenotypic characteristics of molecular neutrophils, which allowed for subsequent ecTrap release assays.

### Neutrophil PMA and bacterial coculture validation

In mimicking human neutrophil differentiation, cells need to be primed prior to a specific response such as oxidative burst, phagocytosis or NET^38^. Once HL-60 cells were differentiated into mature dHL60, we performed kinetic studies to investigate ecTrap release and attempted to validate this release through several different priming methodologies. Our pilot experiments with live imaging cytometry assayed the optimal concentrations of PMA stimulation within the range of 1–10,000 nM. We assayed these conditions and timepoints because previous data indicated that *higher* and *lower* concentrations under *longer* periods of time were able to induce NETs^39^. In our system we attempted to synchronize ecTrap formation by applying PMA (1,000 nM, Fig. 1) and compared the ecTrap morphology through dHL60-bacterial coculture (Fig. S1). Bacterial coculture had the secondary benefit of validating that our dHL60 ecTrap were functional and presenting antimicrobial properties. To determine this, we incubated with a pathogenic strain of gram-negative microbe (*Fusobacterium*) and investigated the microbial viability and the rate of ecTrap *via* elastase reporter. As observed in *ex vivo* and *in vivo* settings^40^, ecTrap were detectable earlier than the 4 hours which is typically required in patient-derived neutrophil NETs by PMA priming^41^. Thus, our pilot PMA and coculture experiments showed that ecTrap was formed from differentiated HL-60 cells *ex vivo* through augmentation with specific bacterial strains or PMA, and that these ecTraps will facilitate inflammation activation and achieve bacterial clearance over 4 hours.

### ecTrap release kinetics and validation

For replicability, we investigated the kinetics to understand the ideal time for ecTrap release (0–6 hours) and attempted to validate through Nanodrop spectrophotometer. However, due to the ecTraps containing a plethora of molecules (such as LL-37) in addition to DNA, the nanodrop results indicated that other methods were necessary. We adapted and performed a previously published NET isolation protocol^24^ and ran the resultant extracts on agarose gel (1.0% w/vol, 100 V, Fig. S7C). Even with our validation and optimization, the replicates seen in Figure S7C indicated the experimental variability, and only samples appearing to have genomic DNA were utilized for further assays.

We sought additional validations of ecTrap DNA isolation by comparing HL-60, dHL60, and ecTrap DNA via scanning electron microscopy (SEM; Fig. 1c). From our ecTrap samples, the only group demonstrating thread- and web-like structures was obtained from the isolation layer – acquired from ecTraps adherent to the culture flask after PBS wash – while other phase layers of the high centrifugation protocol failed to demonstrate these patterns. The presence of lipid bilayers in SEM images of ecTraps could also be vesicles, which are commonly known to be released by neutrophils. After verifying DNA isolation morphologically, we quantified DNA degradation via DNase assay and Agilent High Sensitivity gel. Specifically, DNase was applied to the isolated ecTrap samples and compared to DNase free isolates (Fig. 1e). When we applied DNase to the material released, the signal diminished below the levels of DNA standards shown at 35 and 10,380 bp.

In addition to morphology and DNA existence, we investigated intracellular indicators of the NET release pathway. It is known that prior to NET release, mitochondrial superoxide is formed along with reactive oxygen species (ROS). As such, we evaluated our cells by MitoSOX-red staining of mitochondrial superoxide (Fig. 1d for representative shift in population, S1B). At 2.5 hours, our results showed a peak of ROS formation leading to increased staining of MitoSOX. Quantitatively, stimulation increased the number of oxygen species which allowed the separation of cells into two groups: MitoSOX^Pos^ and MitoSOX^Neg^, with MitoSOX^Pos^ representing the producers of high amounts of ecTraps. Comparatively, dHL60 in PMA were 75.23% positive for MitoSOX versus 14.98% in PBS, while undifferentiated cells in PMA showed 23.05% positive versus 10.89% in PBS. These early events preceded ecTrap formation and were repeated through flow cytometry replicates.

Thus, we validated early ecTrap formation events that preceded DNA release; morphologically compared DNA isolation protocols between HL-60, dHL60, and ecTrap; and quantified the amount of DNA within our ecTrap samples through DNase degradation. To us, after the extensive amount of validation steps, the ecTrap isolate groups clearly present with characteristic morphology and readings of NET DNA. Collectively, we defined a method to differentiate HL-60 to dHL60, and to produce and isolate ecTraps for downstream analysis. Following our validation protocols, we proceeded to extract DNA/RNA/protein from our three groups for proteomics and genomics sequencing.

### ecTrap proteome data and validation

To determine the novelty and useability of proteomic data, analysis was conducted to compare ecTrap proteomes to our undifferentiated whole-cell HL-60 and dHL60. Per condition, 1 × 10^7^ cells were used. The analysis of proteomics data revealed significant differences on the ecTrap groups compared to HL-60 and dHL60 in expression levels of proteins known to be associated with neutrophil function and NETs. To ensure the protein content was from ecTrap and not from cells, our system allowed us to investigate proteins that were exclusive to ecTrap groups and not the cell groups. Principal component analysis showed that ecTraps presented unique clusters when compared to cell proteins (Fig. S4). Out of initial 2,403 proteins quantified in ecTraps, 316 proteins were enriched when compared to dHL60 and HL-60 cells (Fig. S5). Although the isolated ecTraps presented with unique and overlapping sequencings, this group was the only one to demonstrate enrichments for classic pathways for NET formation, confirming our previous validations. In line with the specific protein signatures, differentiated and undifferentiated cells showed reduced numbers of NET markers and presented the highest overlapping rates with pathways related to extracellular exosomes, mitochondrial metabolism, mitochondrial nucleoid, and extracellular matrices (Fig. S5).

To establish sample preparation efficiency before database submission, ecTrap samples were separated into Benzonase and non-Benzonase groups before shotgun proteomic analyses. Namely, one set of samples were directly processed using the established method to generate tryptic peptides for LC-MS/MS analysis^27^, while the other set of samples were treated with Benzonase to degrade all DNA and RNA contents prior to tryptic digestion. Because Benzonase is able to cleave the condensed nucleic acid component in collected ecTrap, we monitored its activity to subsequently release proteins that are tightly bound to the nucleic acids, thus enhancing proteomic mapping^42^. Without Benzonase treatment, the abundance of protein levels was moderate with 1,711 proteins identified. Whereas after Benzonase treatment an increased abundance of proteins was found, with identification of 2,364 proteins (Fig. 2a). To further validate the enrichment of ecTraps by our protocol, we compared protein functions in our study to those previously associated with NETs (Fig. 2b)^8^. The resultant proteome size spans higher than five orders of magnitude, and contains proteins known to be associated with NETs as well as novel proteins.

Functionally, the 100 most abundant proteins identified in our proteome analysis included a classic NET enzyme, Myeloperoxidase (MPO), in high abundance. This was also evident for the calcium and zinc-binding proteins known to be released by blood neutrophils in humans after migrating to the site of inflammation, calprotectin (S100A8 and S100A9), which play an important role in the regulation of inflammation and immune response^43^.

The Label Free Quantitation (LFQ) intensities correlate well within the same treatment groups (Fig. S6), showing consistent reproducibility of a NET proteome profile. Overall, 2,280 proteins were common within the ecTrap groups, and 126 proteins have more than two-fold difference between the two groups by statistical analysis (Permutation FDR 0.05, Fig. 2c). Histone proteins, cathepsin G, S100A8, S100A9, and azurocidin have more than two-fold increase in the Benzonase-treated group, indicating their tight association with the condensed nucleic acid component in ecTraps. Upon functional analysis of the 101 proteins with at least two-fold increase in the Benzonase-treated group, we found that while a fraction of the proteins is involved in leukocyte activation and neutrophil degranulation (FDR = 7.15E-5), another group of proteins related to RNA binding (FDR = 0.0143) was also enriched (Fig. 2d). We attempted to extract pure RNA from ecTraps for additional data submissions but were not able to isolate detectable levels. However, the proteomic findings indicate that the condensed nucleic acid component from ecTrap may not only be the chromosome scaffold, but also includes cellular RNAs.

**Fig. 4:**
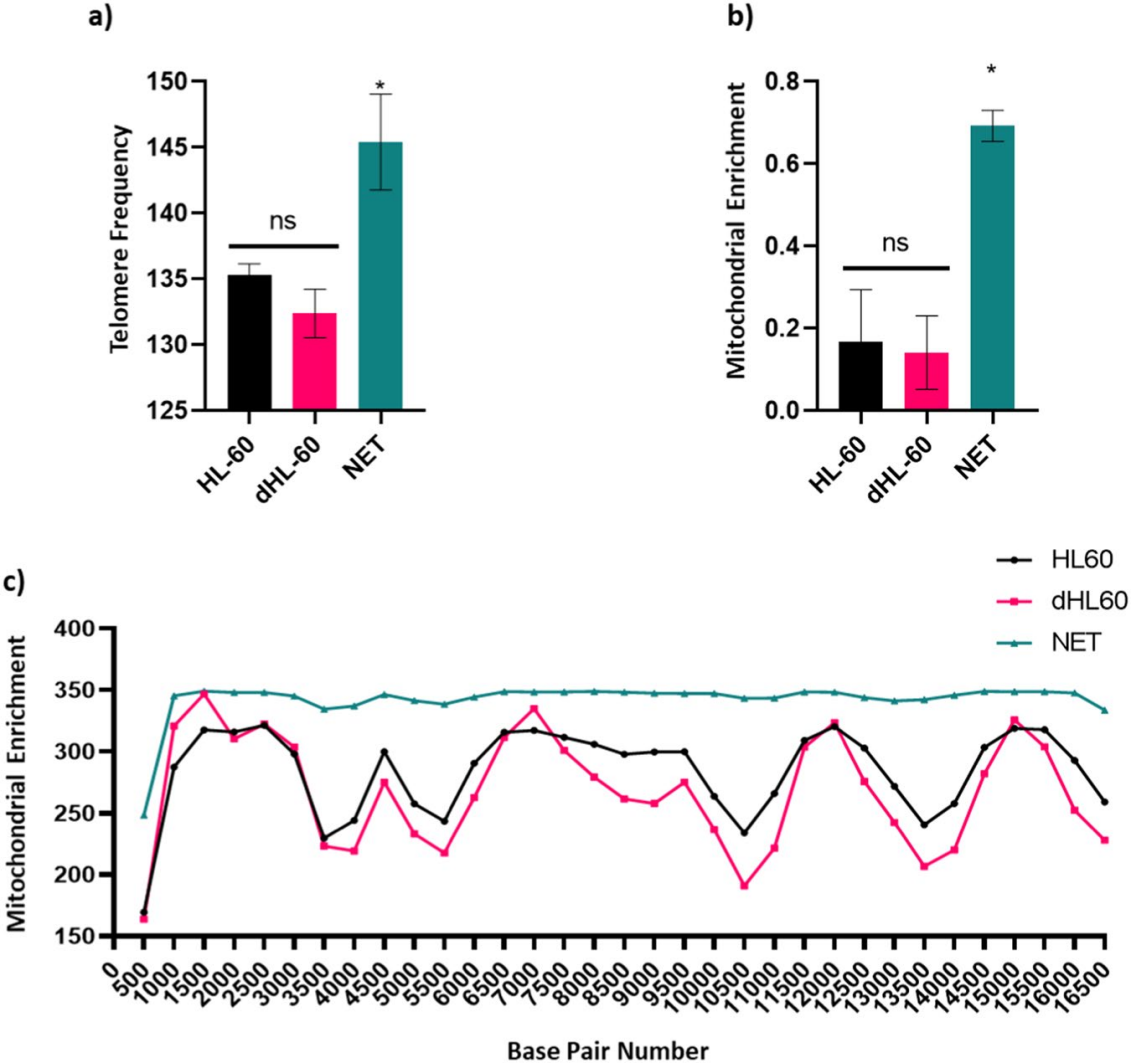
Genomic Enrichment of ecTRapRegions. (**a**) Comparison of telomere counts (TTAGGGTTAGGG) across all chromosomes for each sample (ANOVA, p < 0.05). (**b**) Normalized mitochondrial enrichment quantification from two sequencing runs (n = 2 per sample, ANOVA, p < 0.05). (**c**) Mitochondrial enrichment by position number (sliding window size = 500 nts).

Given the abundance of novel proteins, we sought out alternative data organization methods for pathway enrichment analyses to validate our subsequent genomic sequencing. As such, we organized the proteomic score by peptide spectrum matches (PSM) and input the resultant protein hierarchy into the panther gene ontology pipeline (http://www.pantherdb.org/), which demonstrated pathways associated with positive regulation of neutrophil degranulation, cytosol, mitochondrial proteins, RNA binding molecules, and human telomerase reverse transcriptase activation. We validated these results by measuring mitochondrial DNA and the amount of telomeric sequence repeat TTAGGG from our genomics sequencing (Fig. 4b), which is reflected in the data deposited.

### Neutrophil Extracellular Trap genomic data and validation

To interrogate the genomic sequence of the scaffold DNA released by dHL60, we utilized Illumina’s NexteraXT library prep kit and sequenced ecTrap genome on the NovaSeq 6000 platform. Coverage for isolated Nets were compared to undifferentiated cells and differentiated cells (36X, 45X, and 47X respectively).

The resultant fastq files were input into the CLC Workbench (v11) software, trimmed by 15 nt on the 3′ end to remove primers, trimmed for q-scores <25, mapped to the human genome (hg38, CLC v12), checked for normal human GC content, and comparatively scanned using a sliding window analysis^44^ which was normalized to each sample’s chromosome. Unmappable read files were not used in our analysis.

A simple way to determine whether the datasets submitted to NCBI were different from each other was to determine the existence of genomic enrichment/depleted regions. To that end, a sliding window analysis was conducted for every 20, 100, 500, and 5,000nt, with the 500nt represented for mitochondrial DNA^45^-^47^ (Fig. 4d) and the 5,000nt results represented for whole genome analysis (Fig. 3, S2). After the genome-wise scan was conducted, comparative analyses were made for each possible grouping of samples using a cut-off of 1.5-fold enrichment or 1.5-fold depletion. From this the ecTrap sequence data contained 23,488 regions that were differentially expressed from the other datasets (Figs. S2, S3F). Moreover, the ecTrap sample is quantitatively different based on ROIs, and qualitatively different based on other analyses. Together, these data validate our NETome model and data submission, and verify the submission of neutrophil genomic information^9^,^10^,^30^,^41^,^45^-^50^.

## Supplementary information

SUPPLEMENTARY FIGURE

## Data Availability

No custom code was used, and all analysis was performed using standard software.
