# Supplementary material for: NETome: A model to Decode the Human Genome and Proteome of Neutrophil Extracellular Traps
Source: Sci Data. 2022 Nov 16;9:702. doi: 10.1038/s41597-022-01798-1 (PMC9668915; doi:10.1038/s41597-022-01798-1)
Supplement: Supplementary file 1 — SUPPLEMENTARY FIGURE [file 41597_2022_1798_MOESM1_ESM.pdf]

Table of Contents

Supplementary Figure 1. HL-60 differentiation with comparative ecTrap formation..................2
Supplementary Figure 2. Circos plot of genome-wide scan...3
Supplementary Figure 3. Comparisons using the 5,000nt sliding window between individual samples and averaged sample enrichments. 4
Supplementary Figure 4. Comparison of eccTrap to differentiated and undifferentiated cells....5
Supplementary Figure 5. Comparison of enriched proteins in ecTraps.....................................6
Supplementary Figure 6. Correlation analysis of the six ecTrap samples.................................7
Supplementary Figure 7. Growth and differentiation kinetics of HL-60 cells into dHL60; agarose gel validation of ecTrap isolates...8
Supplementary Figure 8. Giemsa and DAPI HL-60, dHL60, and ecTrap...................................9

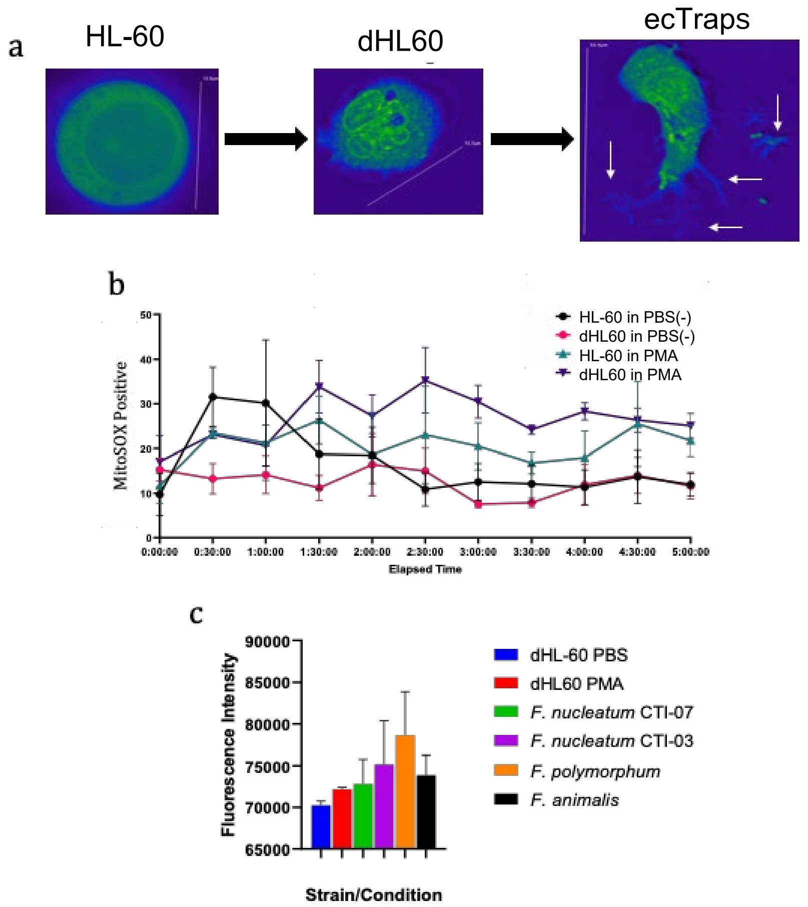

Supplementary Figure 1. HL-60 differentiation with comparative ecTrap formation. (a) Holotomographic microscopy images, digitally stained based on RI (refractive index) confirms the differentiation of HL-60 cells to neutrophils (dHL60) and successful release of ecTraps after coincubation with different strains of bacteria. Arrows point to dHL60 extracellular DNA or bacterial extracellular DNA biolayer. Morphology of partially intact nucleus mimics ecTrap image seen in Figure 1. **(b)** Quantification of flow cytometry utilizing MitoSOX stain under different conditions of PMA or PBS. Maximum dHL60 ROS generation is seen at 2.5 hours which precedes ecTrap formation (n=3, PMA final concentration 1,000 nM). **(c)** Multiwell quantification of neutrophil elastase (NE) through fluorescent activity after coculture with Gram negative *Fusobacterium* strains: *polymorphum, animalis,* and *nucleatum* substrains CTI-03 and CTI-07 or by PMA incubation (n=1 experiment represented).

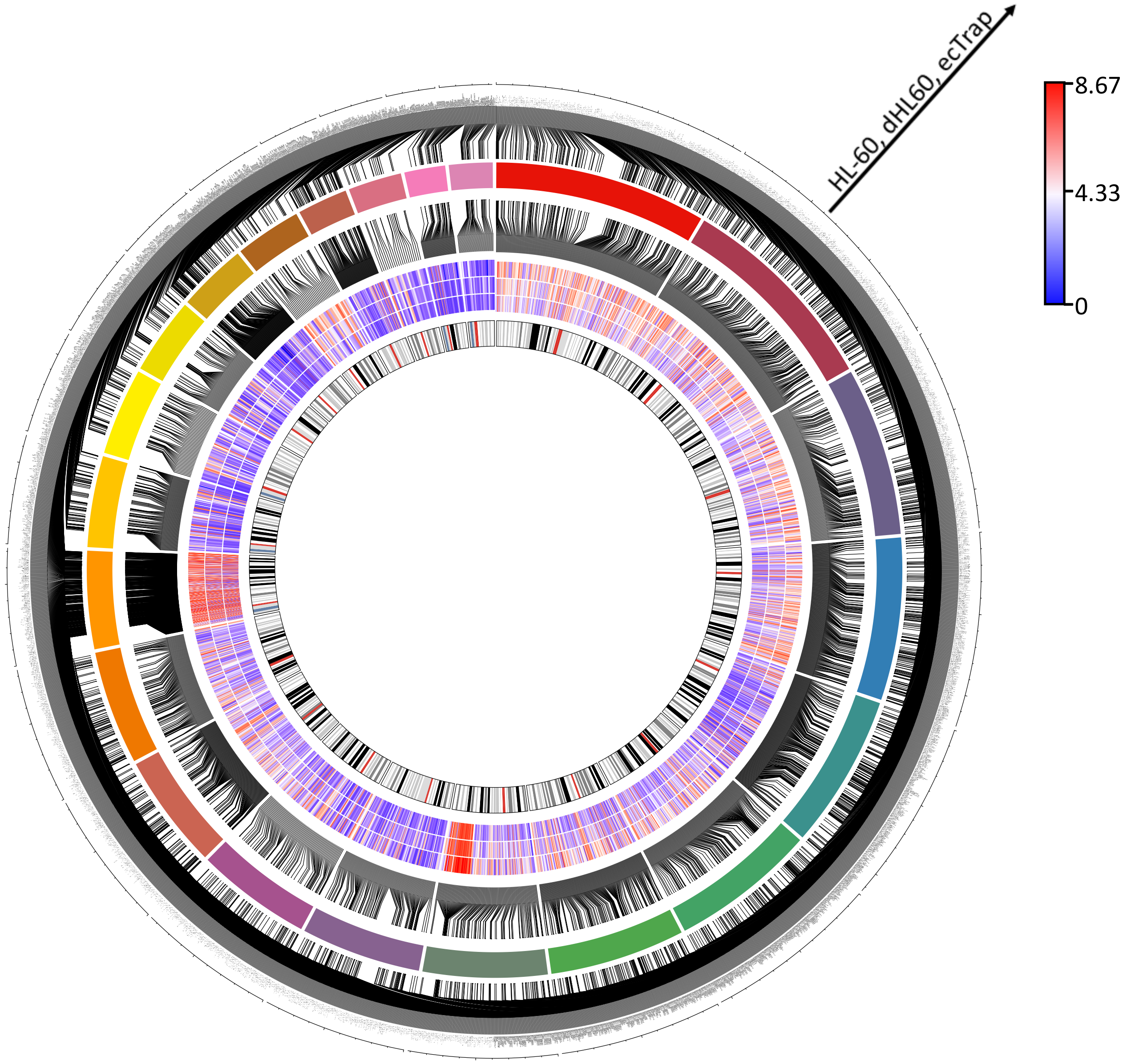

Supplementary Figure 2. Circos plot of genome-wide scan. Genes shown were generated from the 25,488 regions that exhibited consistent 1.5-fold enrichment or depletion in the ecTrap sample relative to HL-60 and dHL60. Enrichment/depletion levels were binned into quantiles ranging from 0 to 8.67 for aesthetic reasons. Table of gene names is available upon request.

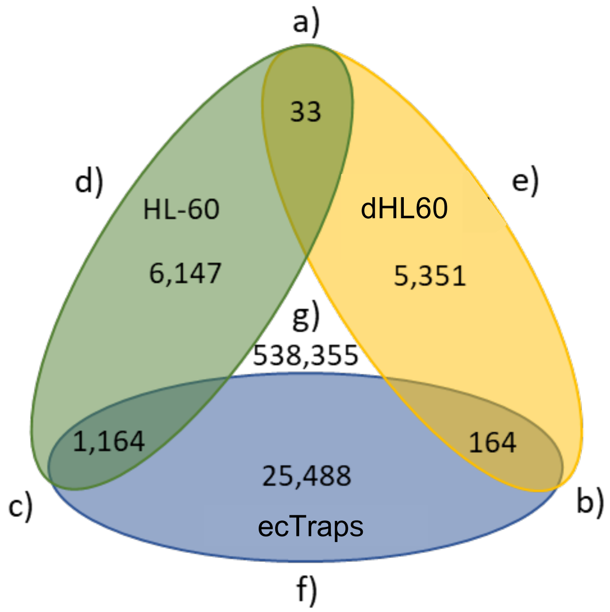

Supplementary Figure 3. Comparisons using the 5,000nt sliding window between individual samples and averaged sample enrichments. **(a-c)** Averages of the two adjacent samples were compared to the opposite sample's enrichment/depletion. **(d-f)** Single sample compared to the unaveraged grouping of the other two samples. Namely, consistent enrichment/depletion over both samples was required. **(a)** Average of HL-60 and dHL60 samples showed enrichment/ depletion in 33 regions over ecTrap samples. **(b)** Average of ecTraps and dHL60 samples showed enrichment/depletion in 164 regions over HL-60 sample. **(c)** Average of ecTraps and HL-60 samples showed enrichment/depletion in 1,164 regions over dHL60 sample. **(d)** HL-60 sample showed consistent enrichment/depletion in 6,147 regions when compared to the grouping of dHL60 and ecTraps. **(e)** dHL60 sample showed consistent enrichment/depletion in 5,351 regions when compared to both, HL-60 and ecTrap. **(f)** ecTraps are enriched/depleted in 25,488 regions when compared to both, HL-60 and dHL60. **(g)** There are 538,355 regions that show no consistent enrichment or depletion among any sample when compared to the grouping of the other two.

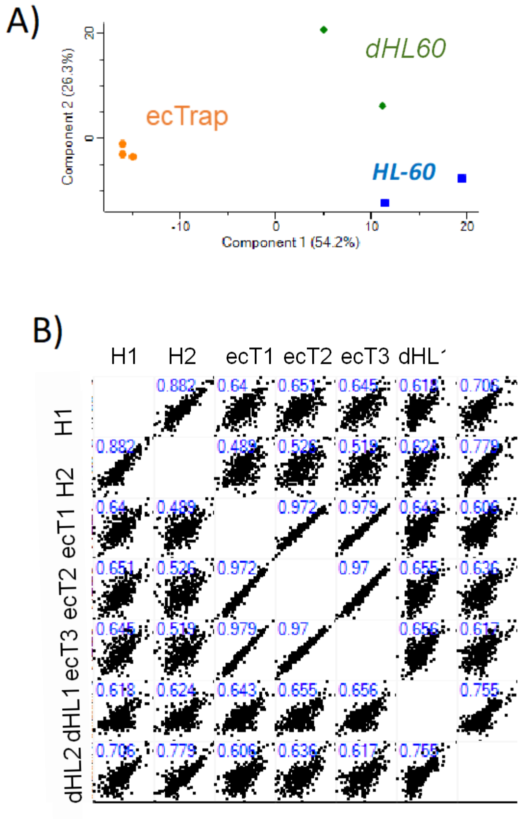

Supplementary Figure 4. Comparison of ecTraps to differentiated and undifferentiated cells. (A) proteomic principal component analysis demonstrating clusters of ecTrap groups in comparison to dHL60 and HL-60 cells. **(B)** Pearson correlation values between HL-60 (H), dHL60 (dHL) and ecTraps.

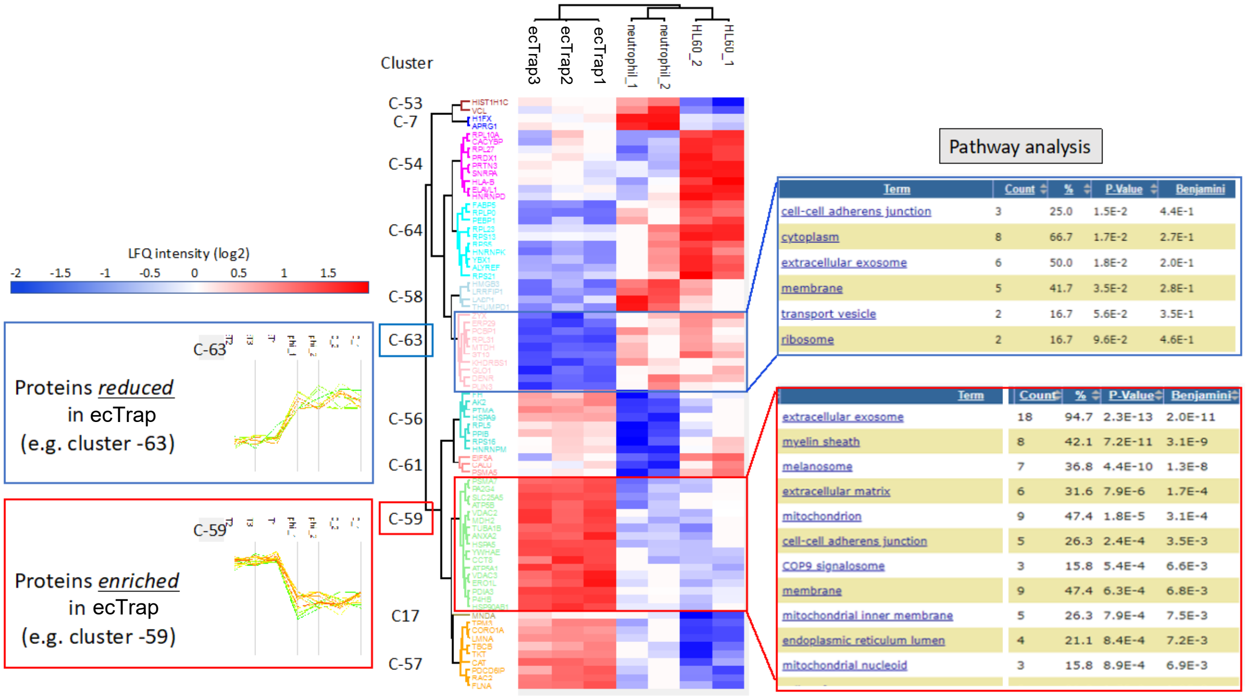

Supplementary Figure 5. Comparison of enriched proteins in ecTraps. In comparison to dHL60 and HL-60 cells, 75 proteins were enriched (downregulation in blue, upregulation in red). Pathway analysis demonstrated top functions involved in proteins enriched in ecTraps.

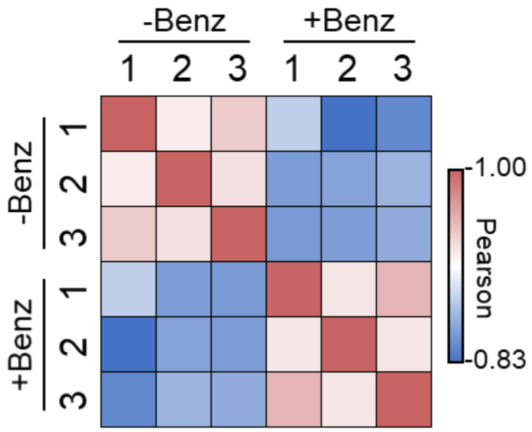

Supplementary Figure 6. Correlation analysis of the six ecTrap samples. Pearson correlation values were color coded as indicated in the scale bar (n = 3 for each group, with or without Benzonase treatment).

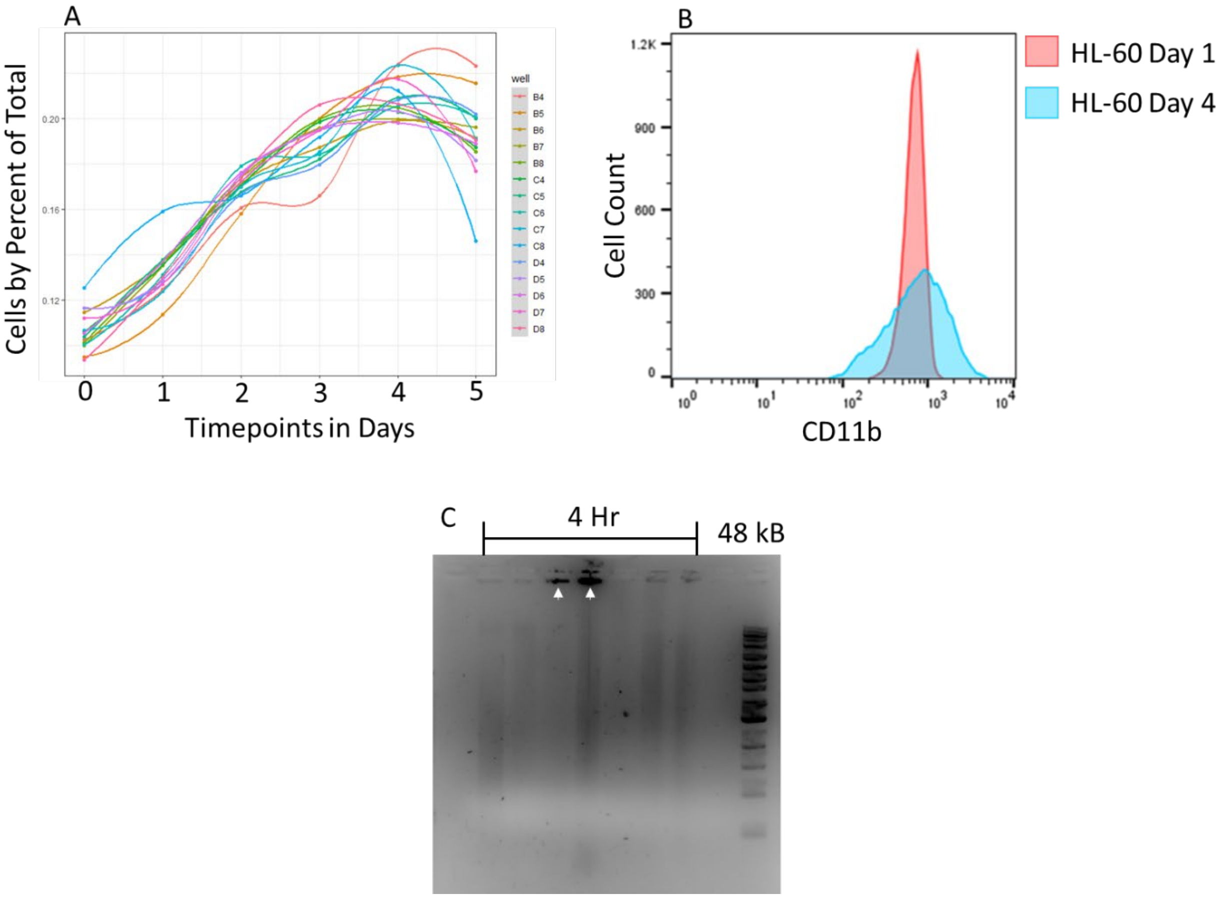

Supplementary Figure 7. Growth and differentiation kinetics of HL-60 cells into dHL60; agarose gel validation of ecTrap isolates. (A) DMSO was added to HL-60 cells, and their growth was monitored over 5 days. Day 5 growth indicated cells undergoing apoptosis which informed day 4 as a reasonable test for maximum differentiation. (B) Flow cytometry of HL-60 cells under DMSO condition after 1 and 4 days. Early differentiation marker CD11b showed an increased cell population in HL-60 cells under DMSO after 1 day versus 4 days, indicating early differentiation at day 1 and late differentiation at day 4. (C). Agarose gel replicates performed on ecTrap-producing cells incubated in 1,000 nM PMA over 4 hours. Arrows point to genomic DNA.

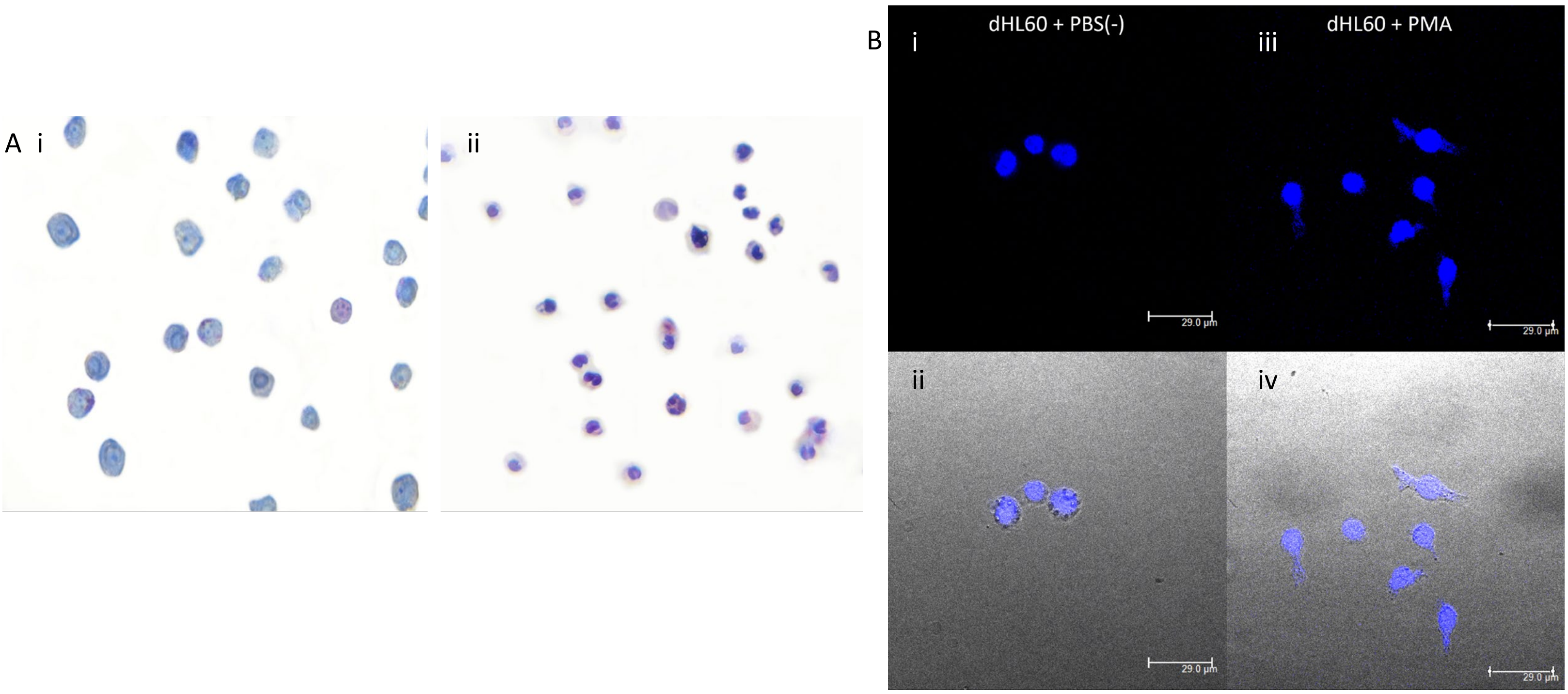

Supplemental Figure 8. Giemsa and DAPI HL-60, dHL60, and ecTraps.A) Giemsa-stained HL-60 cells (i) and after differentiation to dHL60 cells (ii). Morphological changes are representative of polymorphonuclear phenotype. B) DAPI staining of nucleotides after incubation of dHL60 cells with PBS without calcium and magnesium (i; PBS(-)), merged with bright field (ii), or incubated with 1,000nm PMA (iii), merged with bright field (iv).
